# Cost-Effectiveness of Testing for NS5A Resistance to Optimize Treatment of Elbasvir/Grazoprevir for Chronic Hepatitis C in China

**DOI:** 10.3389/fphar.2021.717504

**Published:** 2021-10-15

**Authors:** Jinyu Liu, Yu Zhang, Bin Wu, Sen Wang, David Bin-Chia Wu, Ruxu You

**Affiliations:** ^1^ Department of Pharmacy, Tongji Hospital, Tongji Medical College, Huazhong University of Science and Technology, Wuhan, China; ^2^ Department of Pharmacy, Union Hospital, Tongji Medical College, Huazhong University of Science and Technology, Wuhan, China; ^3^ Department of Pharmacy, Ren Ji Hospital, Shanghai Jiao Tong University School of Medicine, Shanghai, China; ^4^ Department of Infectious Diseases, Huashan Hospital, Fudan University, Shanghai, China; ^5^ School of Pharmacy, Monash University Malaysia, Kuala Lumpur, Malaysia; ^6^ Saw Swee Hock School of Public Health, National University of Singapore, Singapore, Singapore

**Keywords:** cost-effectiveness, baseline testing, resistance-associated polymorphisms, elbasvir/grazoprevir, hepatitis C virus

## Abstract

**Objectives:** Baseline presence of nonstructural protein 5A (NS5A) resistance-associated variants can attenuate the efficacy of new direct-acting antivirals. A potential method to attain the higher efficacy would be to screen for NS5A polymorphisms prior to the initiation of therapy and to adjust the treatment length based on the test results. However, baseline testing adds additional costs and it is unclear whether this would represent a high value strategy for chronic hepatitis C in China.

**Methods:** A hybrid model compared 1) standard 12-weeks treatment (no testing), 2) shortened 8-weeks treatment (no testing), and 3) baseline testing with 12-/8-weeks treatment for those with/without NS5A polymorphisms from a lifetime Chinese health care payer perspective. All model inputs were retrieved from clinical trials and publically available literature. And sensitivity analyses were also conducted to assess the impact of uncertainty.

**Results:** Baseline testing was associated with overall increase in total health care cost of USD 13.50 and in QALYs of 0.002 compared with standard 12-weeks treatment (no testing), yielded in an ICER of USD 6750/QALY gained. Scenario analyses suggested that shortened 8-weeks treatment (no testing) was found to be lower costs and great QALYs compared with other two strategies when the sustained virologic response (SVR) rate increased to 95%. Sensitivity analyses indicated that the results were robust.

**Conclusions:** Our results suggest prior assessment of NS5A sensitivity followed by optimizing treatment duration was an economic strategy. In addition, shortened 8-weeks treatment (no testing) was shown to be dominant with the SVR rate increased to 95%.

## Introduction

Infection with hepatitis C virus (HCV) is a leading cause of liver disease and often presents as a chronic disease with nonspecific symptoms. Worldwide, an estimated 130–170 million people have HCV infection, and China has the most cases of HCV infection, with an estimated 29.8 million people (Hajarizadeh et al., 2013; Han and Liu, 2016). The long-term hepatic impact of HCV infection is highly variable with a spectrum ranging from minimal hepatic inflammation to extensive fibrosis and cirrhosis with or without hepatocellular carcinoma (HCC). Advanced live disease stages significantly affect patients by reducing their quality of life and impairing their work productivity to perform daily activities. Moreover, costing studies on the management of chronic hepatitis C found that without effective treatment, 420,000 new cases of HCV-related cirrhosis and 254,000 new cases of HCV-related HCC would occur over the next 15 years, leading to future treatment costs of 589 million and 611 million dollars, respectively, (Wu et al., 2019; Xie et al., 2019).

Pegylated-interferon (Peg-IFN) and ribavirin (RBV) combination therapy (PR) has been the standard of care for chronic HCV infection. However, treatment with PR has limited efficacy due to low sustained virologic response (SVR) rates (40–70%) (Wu et al., 2019; Xie et al., 2019). Significant side events and contraindications may also accompany the duration of PR treatment, resulting in poor adherence and premature treatment discontinuation. Therefore, novel treatments that have more potent antiviral activity and fewer adverse effects and are eligible and compatible for patients with complex comorbidity in real-world settings are urgently required.

Fortunately, with the advent of direct-acting antivirals (DAAs), the treatment for chronic HCV has reached another milestone and the treatment response is satisfactory in general, which produce high SVR rates, fewer side effect, shorter courses of treatment and improved medication persistence and compliance (Doica et al., 2021). However, there is emerging evidence that therapy over the licensed length of treatment is less likely to be effective in some patients (Itakura et al., 2015; Cloherty et al., 2016). Resistance-associated substitutions (RASs) of HCV in the nonstructural protein 5A (NS5A) region can impair the efficacy of DAA regimens containing NS5A inhibitors. For example, patients with chronic HCV genotype 1b (GT 1b) infection received elbasvir/grazoprevir (ELB/GZR), one commonly used combination therapy, achieves SVR rate of 90.0% over the shortened 8-weeks treatment (Huang et al., 2019), and a higher rate (97.5%) after the standard 12-weeks treatment (Huang et al., 2019; Wei et al., 2019a). Nevertheless, patients resistant to NS5A have a significantly lower cure rate over the 8-weeks treatment (75%) than over the 12-weeks treatment (94.3%) (Huang et al., 2019; Wei et al., 2019b).

A potential method to attain the higher efficacy for these patients would be to screen for NS5A polymorphisms prior to the initiation of therapy and to adjust the treatment length based on the test results. However, baseline testing adds additional costs and it is unclear whether this would represent a high value strategy. In order to assess this, we explore the entire lifetime cost-effectiveness of baseline testing for NS5A RASs in EBR/GZR-treated GT 1b patients in China, with treatment duration optimized to 8 weeks in NS5A-sensitive individuals and 12 weeks otherwise (Chinese Society of Hepatology and Chinese Society of Infectious Diseases, Chinese Medical Association, 2019; Huang et al., 2019; Wei et al., 2019a). We compared blind treatment of all patients with a standard 12-weeks treatment duration, which is the generally recommended treatment length. In addition, a shortened 8-weeks treatment duration was also examined because this strategy is sometimes recommended, particularly for newer DAAs.

## Methods

### Overview

The reporting of the current economic evaluation followed the Consolidated Health Economic Evaluation Reporting Standards (CHEERS) (Husereau et al., 2013). A hybrid model was developed to evaluate the cost-effectiveness of baseline testing for RASs to optimize treatment of ELB/GZR in patients with chronic HCV GT 1b infection in China. The main output of current research was the incremental cost-effectiveness ratios (ICERs) calculated for cost per quality-adjusted life-years (QALYs) for one clinical strategy compared with the others. The model aimed to simulate the patient’s lifespan by dividing it into equal cycles (1 year) to capture relevant costs and consequences of advanced liver-related complications during the treatment period. We conducted the analyses from health care payer (only including direct medical costs) perspectives. A discount rate of 5% was used for costs and QALYs, which was based on the recommendations in the China Guidelines for Pharmacoeconomic Evaluations (Liu, 2020). Three times the per capita gross domestic product (GDP) value of China in 2019 ($30,829) was used as the willingness-to-pay (WTP) threshold. The model was programmed using TreeAge Pro 2019 (TreeAge Software Inc., Williamston, MA, United States ).

### Model Structure

**FIGURE 1:**
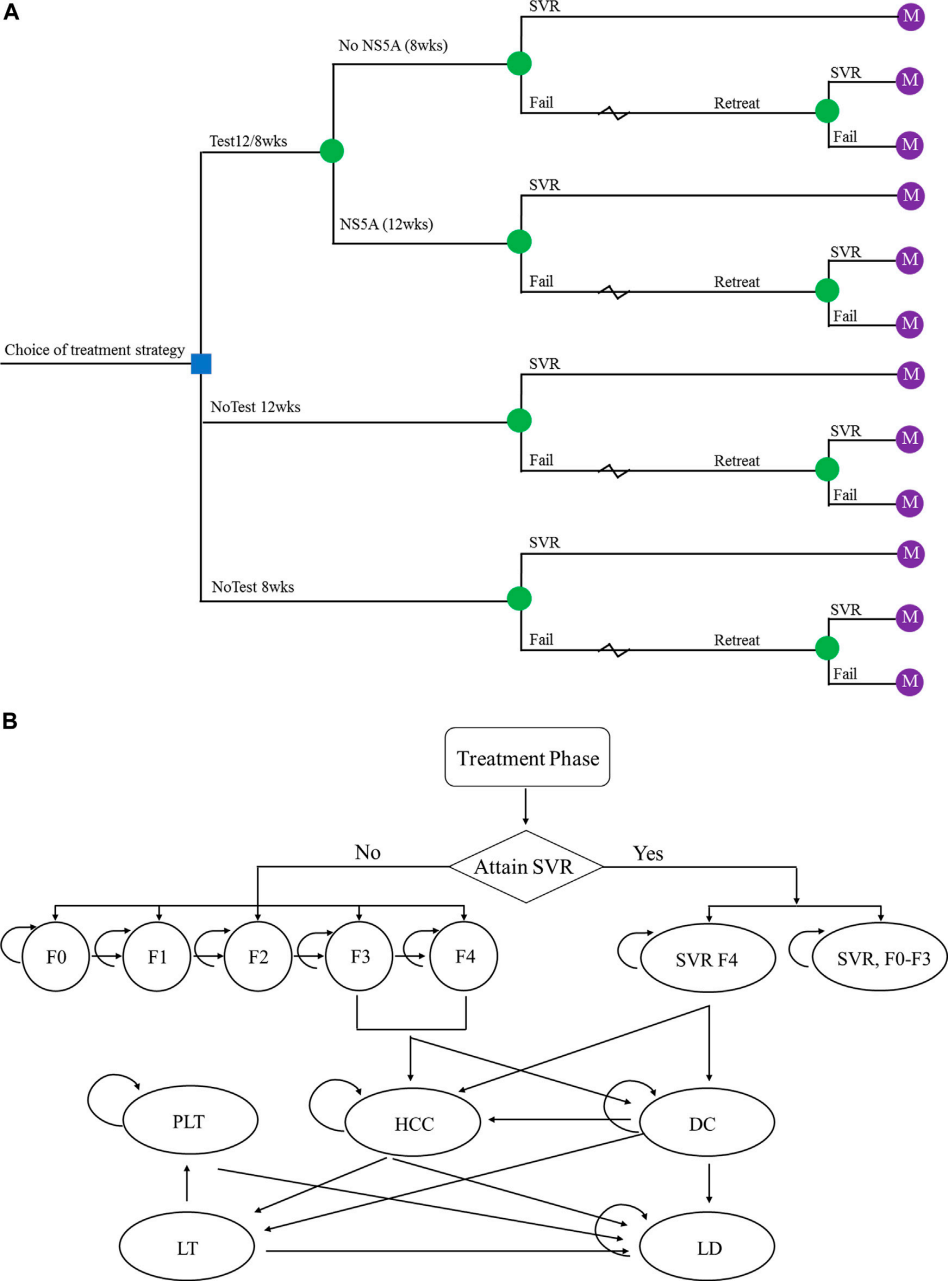
The schematics of **(A)** the decision tree and **(B)** the Markov state transition model. Abbreviations: SVR, sustained virologic response; F0–F4, Metavir fibrosis score; SVR F0-F3, patients diagnosed at F0–F3 fibrosis stage achieved SVR; SVR F4, patients diagnosed at F4 fibrosis stage achieved SVR; DC, decompensated cirrhosis; HCC, hepatocellular carcinoma; LT, liver transplant; PLT, post-liver transplant; LD, liver-related death.

The simplified representation of the model structure is shown in Figure 1. The economic model that combined a decision tree and a Markov cohort model was constructed. A decision tree model was developed to present the three alternative strategies (Figure 1A). In the no-testing strategy, patients were treated for either 12 weeks (standard treatment duration) or 8 weeks (short treatment duration) regardless of NS5A resistance status. In the testing group, patients received 12 weeks treatment duration if NS5A resistance, 8 weeks otherwise. A Markov model was used to simulate the natural history and progression of chronic HCV disease (Figure 1B). All patients entered the Markov model on the basis of their response to treatment and initial liver fibrosis. Each subsequent year, they could progress to a higher METAVIR stage (F0–F4) or, develop decompensated cirrhosis (DC) and HCC. After achieving SVR, it is assumed that patients in stage F0–F3 would not progress to an advanced stage. The progression rates to DC or HCC among patients in stage F4 would be reduced. Those with DC or HCC could receive liver transplantation (LT). In addition, Expected deaths were calculated from the mortality rate of the Chinese general population applied to each stratum of age and sex. Patients with advanced liver-related complications had excess mortality rates. Table 1 shows the key parameters used in the health economics model.

**TABLE 1:** Summary of treatment, epidemiological, cost, and quality-of-life inputs.

	Value	Range	Distribution	References
Efficacy (SVR rates)				
First-line treatment: ELB/GZR				
NoTest12 weeks	0.975	0.960–0.985	Beta	Wei et al. (2019a)
NoTest 8 weeks	0.900	0.835–0.965	Beta	Huang et al. (2019)
Test 12/8 weeks				
NS5A (12 weeks)	0.943	0.924–0.962	Beta	Wei et al. (2019b)
No NS5A (8 weeks)	0.964	0.945–0.985	Beta	Huang et al. (2019)
Retreatment: SOF/VEL/VOX				
NoTest 12 weeks/NoTest 8 weeks	0.973	0.941–0.992	Beta	Bourlière et al. (2017)
Test 12/8 weeks				
NS5A (12 weeks)	0.968	0.930–0.991	Beta	Bourlière et al. (2017)
No NS5A (8 weeks)	0.977	0.916–0.999	Beta	Bourlière et al. (2017)
NS5A resistance prevalence	0.141	0.100–0.190	Beta	Lu et al. (2019)
Annual transition probabilities Fibrosis progression				
F0–F1	0.117	0.105–0.129	Beta	Thein et al. (2008)
F1-F2	0.085	0.077–0.094	Beta	Thein et al. (2008)
F2-F3	0.120	0.108–0.132	Beta	Thein et al. (2008)
F3-F4	0.116	0.104–0.128	Beta	Thein et al. (2008)
F4-DC	0.039	0.031–0.047	Beta	Fattovich et al. (1997)
F4-HCC	0.024	0.019–0.029	Beta	Fattovich et al. (1997)
DC-HCC	0.068	0.054–0.082	Beta	Townsend et al. (2011)
DC-LT	0.023	0.018–0.028	Beta	Townsend et al. (2011)
DC-LD	0.104	0.083–0.125	Beta	(Hui et al., 2002; Liu et al., 2012; Wang et al., 2012)
HCC-LT	0.040	0.032–0.048	Beta	Townsend et al. (2011)
HCC-LD	0.520	0.416–0.624	Beta	Hui et al. (2002); Liu et al. (2012); Wang et al. (2012)
LT-LD	0.194	0.155–0.233	Beta	Kuwabara et al. (2015)
PLT-LD	0.049	0.039–0.059	Beta	Kuwabara et al. (2015)
SVR F4-DC	0.003	0.002–0.004	Beta	Dienstag et al. (2011)
SVR F4-HCC	0.006	0.005–0.007	Beta	Dienstag et al. (2011)
Costs (2019 USD)				
Resistance test costs	80	60–100	Uniform	—
Treatment-related costs (Monthly)				
ELB/GZR	317.930	254.344–381.516	Uniform	—
SOF/VEL/VOX	4,783.359	3,826.687–5,740.030	Uniform	—
Annual Health states costs				
F0-F3	990.286	669.550–1,311.022	Gamma	Chen et al. (2016)
F4	2,818.588	998.925–4,638.252	Gamma	Chen et al. (2016)
DC	6,277.569	3,814.274–8,740.864	Gamma	Chen et al. (2016)
HCC	13,250.605	9,529.204–16,973.085	Gamma	Chen et al. (2016)
LT	62,796.168	50,236.934–75,355.401	Gamma	Wei et al. (2013)
PLT	9,198.961	7,359.169–11,038.753	Gamma	Wei et al. (2013)
Relative costs in post-SVR F3-F4	0.709	0.592–0.855	Lognormal	Manos et al. (2013)
Utilities				
Utilities with disease stages				
F0-F1	0.878	0.751–0.985	Beta	Chhatwal et al. (2015); Chahal et al. (2016); Chen et al. (2016); Chen and Chen (2017)
F2-F3	0.863	0.701–0.985	Beta	
F4	0.792	0.670–0.907	Beta	
DC	0.713	0.517–0.837	Beta	
HCC	0.685	0.532–0.821	Beta	
LT	0.663	0.563–0.800	Beta	
PLT	0.773	0.636–0.850	Beta	
SVR F0-SVR F1	0.928	0.806–1.000	Beta	
SVR F2	0.911	0.791–1.000	Beta	
SVR F3	0.893	0.766–1.000	Beta	
SVR F4	0.850	0.722–0.955	Beta	
Discounts				
Costs	0.05	0.00–0.08	Triangular	Liu (2020)
Utilities	0.05	0.00–0.08	Triangular	Liu (2020)

†Abbreviations: SVR, sustained virologic response; ELB/GZR, elbasvir/grazoprevir; SOF/VEL/VOX, sofosbuvir/velpatasvir/voxilaprevir; NS5A, nonstructural protein 5A; F0–F4, Metavir fibrosis score; SVR F0-F4, patients diagnosed at F0-F4 fibrosis stage achieved SVR; DC, decompensated cirrhosis; HCC, hepatocellular carcinoma; LT, liver transplant; PLT, post-liver transplant; LD, liver-related death.

### Patient Characteristics

The target population of the analysis was defined as treatment naïve patients with chronic HCV GT 1b infection in China. Enrolled patients had a mean of 55 years of age and 52.0% of them were male, which was determined based on the previous population-based effectiveness study. The distribution of patients with chronic hepatitis C by fibrosis stages was as follows: F0 0.8, F1 45.5, F2 41.3, F3 9.9, and F4 2.5% based on a recent Chinese study (Li et al., 2014). According to Lu et al. (2019), the prevalence of NS5A resistance in the Chinese population was reported at 14.1%.

### Treatment Strategies and Clinic Inputs

Three strategies were assessed based on the response to NS5A resistance testing and treatment duration. In the “no NS5A-testing strategy”, NS5A resistance testing was not conducted, and all patients were treated with ELB/GZR for either standard 12-weeks treatment duration (NoTest 12 weeks) or short 8-weeks treatment duration (NoTest 8 weeks). In the “NS5A-testing strategy”, testing for NS5A resistance was performed before treatment was determined; patients who tested NS5A-resistant received ELB/GZR 12 weeks, and those who tested NS5A-sensitive received ELB/GZR 8 weeks (Test 12/8 weeks).

We assumed ELB/GZR as first-line therapy for chronic HCV GT1b infection that is administered once daily using an oral fixed-dose combination of 50 mg elbasvir and 100 mg grazoprevir, as per recent Chinese clinical guidelines (Chinese Society of Hepatology and Chinese Society of Infectious Diseases, Chinese Medical Association, 2019). Patients who failed to achieve SVR in the treatment arm would be retreated with sofosbuvir/velpatasvir/voxilaprevir (SOF/VEL/VOX) as a salvage regimen administered once daily (Chinese Society of Hepatology and Chinese Society of Infectious Diseases, Chinese Medical Association, 2019). SOF/VEL/VOX is also an NS5A inhibitor-containing regimen containing 400 mg sofosbuvir, 100 mg velpatasvir, and 100 mg voxilaprevir. SVR data for 12 weeks of ELB/GZR were derived from an integrated analysis of data from international multicenter clinical trials mainly in Chinese participants (Wei et al., 2019a; Wei et al., 2019b), and short 8-weeks treatment efficacy sourced from an open-label, randomized, active control trial (EGALITE) in Taiwan (Huang et al., 2019). As clinical efficacy data about SOF/VEL/VOX in the Chinese context was not available, we utilized data collected from two phase clinical trials (POLARIS-1 and POLARIS-4) in other countries (Bourlière et al., 2017).

### Transition Probabilities

Data on the fibrosis progression rates from F0 to F4 were collected from a meta-analysis and meta-regression (Thein et al., 2008), and progression rate F4 to DC and HCC were modeled based on retrospective follow-up studies of HCV-infected patients (Fattovich et al., 1997). The annual probabilities from DC to HCC and LT and from HCC to LT were retrieved from a recent systematic literature review (Townsend et al., 2011). Patients with F4 who achieved SVR could still develop advanced complications, but at a decreased rate compared to those not achieving SVR (RR = 0.0857 and 0.2400 for DC and HCC, respectively) (Dienstag et al., 2011). Probabilities of HCV-related death were taken from published Chinese (Hui et al., 2002; Liu et al., 2012; Wang et al., 2012) and Japanese studies (Kuwabara et al., 2015) on liver-related mortality for the DC, HCC, LT, and post-liver transplant (PLT) disease states. For non-HCV-related causes of death, mortality was derived from 2019 life table for Chinese population by age (National Health Committee of the People's Republic of China, 2019).

### Costs

Direct medical costs consisted of drug costs, NS5A resistance testing, and health state costs. The costs of ELB/GZR and SOF/VEL/VOX were determined by the manufacturers. Cost for NS5A resistance testing was estimated based on patient records in local hospitals. Annual costs associated with F0–F4, DC, and HCC were based on a recent survey of patients with chronic HCV infection in China (Chen et al., 2016); relevant costs of liver-related and other laboratory tests, procedures, medications, and hospitalizations were included. Costs were decreased in F0-F4 patients achieving an SVR (Manos et al., 2013). Calculated and updated LT surgery cost and PLT cost with China Liver Transplant Registry and China Advisory Board (Wei et al., 2013). All costs were converted to USD using official exchange rates as of 2019 (1 USD = 6.8985 CNY) and were inflated to 2019 prices using China Consumer Price Index (CPI) (National Bureau of Statistics of China).

### Utilities

Because utility values on the disease states for Chinese patients were limited, we extracted the values from previously published studies (Chhatwal et al., 2015; Chahal et al., 2016; Chen et al., 2016; Chen and Chen, 2017). Patients who achieved SVR were assumed to receive a utility increment with living in the fibrosis stages.

### Analysis

We performed base-case, deterministic (one-way, two-way) sensitivity, probabilistic sensitivity and scenario analyses. One-way sensitivity analysis was undertaken by varying one parameter in a range between an upper and lower bound while all other parameters were kept constant. Two-way sensitivity analysis was performed to identify the optimal alternative for each combination of values of the two variables. Probabilistic sensitivity analysis was conducted to evaluate the impact of the joint uncertainty surrounding the model variables using Monte-Carlo simulations (1,000 simulations and 10,000 trials per simulation). We also examined different scenarios in which: 1) target patients with compensated cirrhosis entered the model in the F4 state only, 2) target patients with noncirrhosis patients entered the model in the F0–F3 states, 3) drug costs of second-line reduced by 85%, 4) drug regimen for second-line therapy switched to glecaprevir/pibrentasvir (GLE/PIB), and 5) SVR rate of NoTest 8 weeks increased to 95%.

## Results

### Base Case Analysis

Table 2 presented the total health care costs, the number of clinical events, QALYs and ICER estimated by the model. Compared with No Test 12 weeks (mean cost USD 3884.73; mean effect 13.402 QALYs), No Test 8 weeks was shown to be dominated with higher costs and less health benefits. However, Test 12/8 weeks (mean cost USD 3898.23; mean effect 13.404 QALYs) was associated with overall increase in total health care cost of USD 13.50 and in QALYs of 0.002, yielded in an ICER of USD 6750/QALY gained. Besides, both NMB and NHB further indicated similar conclusions to the ICER.

**TABLE 2:** Summary of the cost and outcome results in base-case analysis.

	Base case values			Incremental values	
	Test 12/8 weeks	NoTest 8 weeks	NoTest 12 weeks	Test 12/8 weeks *vs.* NoTest 12 weeks	NoTest 8 weeks *vs.* NoTest 12 weeks
Patient cost over lifetime (2019 USD)	3,898.23	4,686.30	3,884.73	13.50	801.57
Clinical outcomes (%)					
Decompensated cirrhosis	1.06	1.03	0.76	0.30	0.27
Hepatocellular carcinoma	0.78	1.07	1.02	–0.24	0.05
Liver transplantation	0.02	0.07	0.06	–0.04	0.01
Live-related death	0.55	0.70	0.63	–0.08	0.07
QALYs	13.404	13.400	13.402	0.002	–0.002
ICER (USD/QALY gained)	—	—	—	6,750	–400785
NHB	—	—	—	0.002	–0.028
NMB	—	—	—	48.158	–863.228

†Abbreviations: USD, United states Dollars; QALYs, quality-adjusted life years; ICER, incremental cost-effectiveness ratio; NHB, net health benefit; NMB, net monetary benefit.

### One-Way Sensitivity Analysis

**FIGURE 2:**
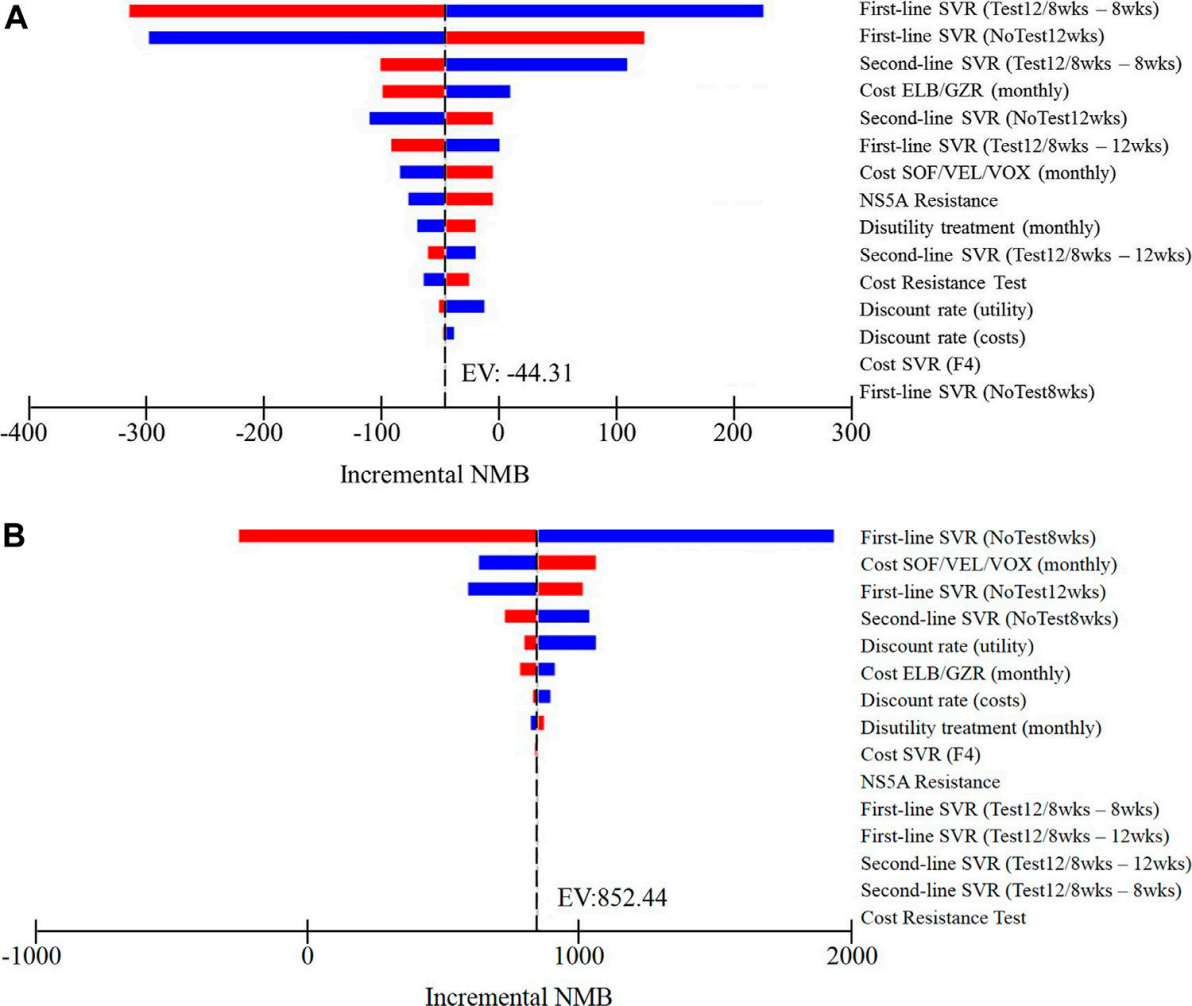
One-way sensitivity analyses show the lower and upper values for the cost-effectiveness ratio of **(A)** Test 12/8 weeks strategy vs. NoTest 12 weeks strategy and **(B)** NoTest 8 weeks strategy vs. NoTest 12 weeks strategy for each parameter.

The results of one-way sensitivity analysis of NoTest 8 weeks and Test 12/8 weeks vs. NoTest 12 weeks were presented as tornado plots showing the influences of extreme variations in each parameter (Figure 2). The study demonstrated that the most impactful parameters were the SVR rates over a standard 12-weeks treatment and a shortened 8-weeks treatment and the cost of first-line treatment and retreatment.

### Two-Way Sensitivity Analysis

**FIGURE 3:**
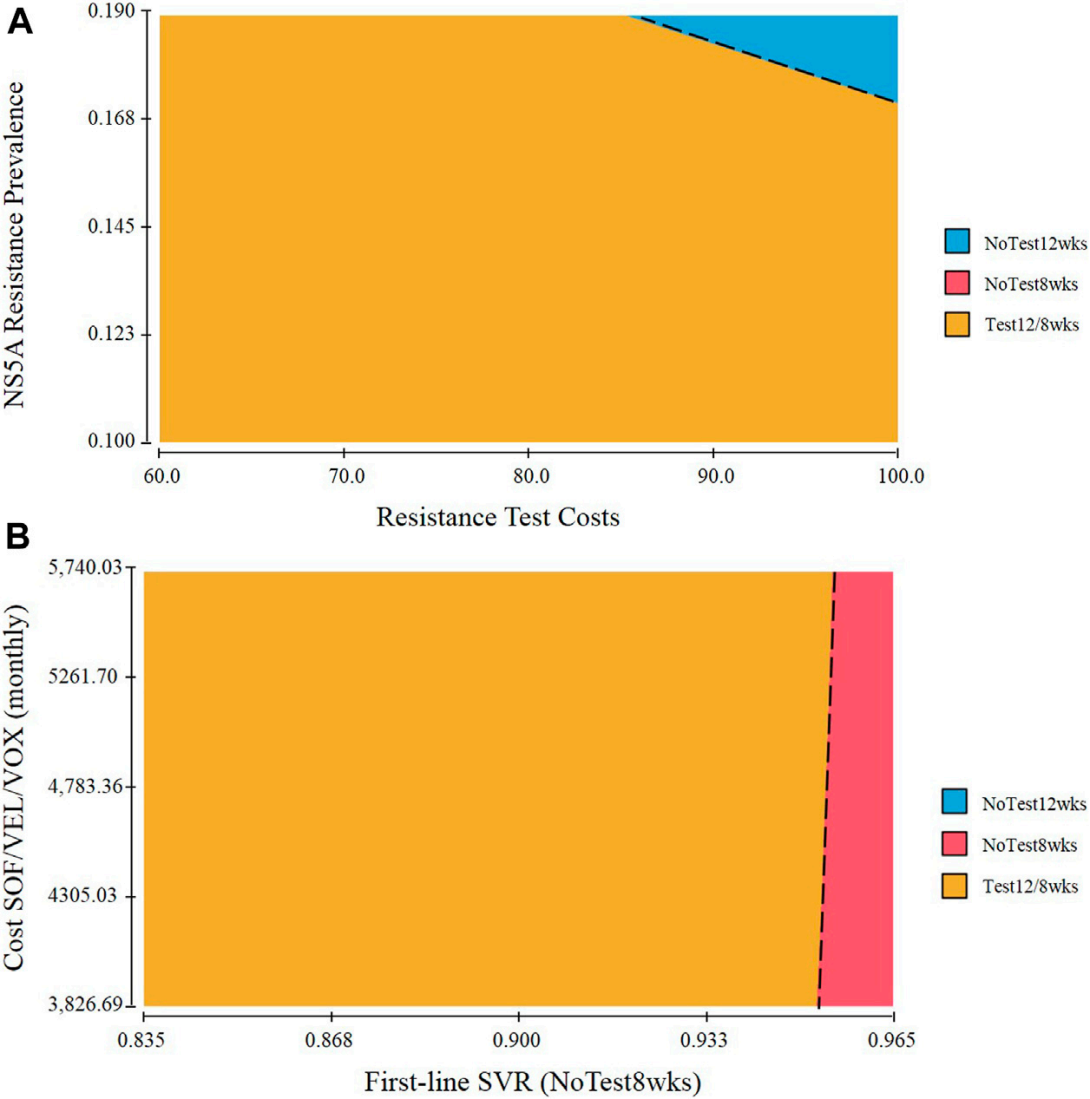
Two-way sensitivity analysis of **(A)** NS5A resistance prevalence and resistance test costs. The dot line represents points at which performing NoTest 12 weeks and Test 12/8 weeks are equally cost effective. Combinations of input variables below and to the left of the line denote circumstances in which Test 12/8 weeks is the cost-effective approach and above and to the right favor NoTest 12 weeks **(B)** The probability of SVR in the NoTest 8 weeks strategy and the cost of second-line treatment.

A two-way sensitivity analysis incorporating NS5A resistance prevalence and resistance test costs was explored (Figure 3A). Most of the variable value combinations recommend the Test 12/8 weeks strategy. However, only when the population with a higher rate of resistance-associated substitutions and very high costs of resistance test, the preferred strategy is NoTest 12 weeks. The probability of SVR in the NoTest 8 weeks strategy and the cost of second-line treatment had a high impact on results in the one-way sensitivity analysis. We therefore performed a two-way sensitivity analysis on these variables (Figure 3B). The Test 12/8 weeks treatment was the best strategy in most cases. The NoTest 8 weeks treatment could be recommended in a few situations (a very high probability of SVR rate over shortened 8-weeks treatment duration).

### Probabilistic Sensitivity Analysis

**FIGURE 4:**
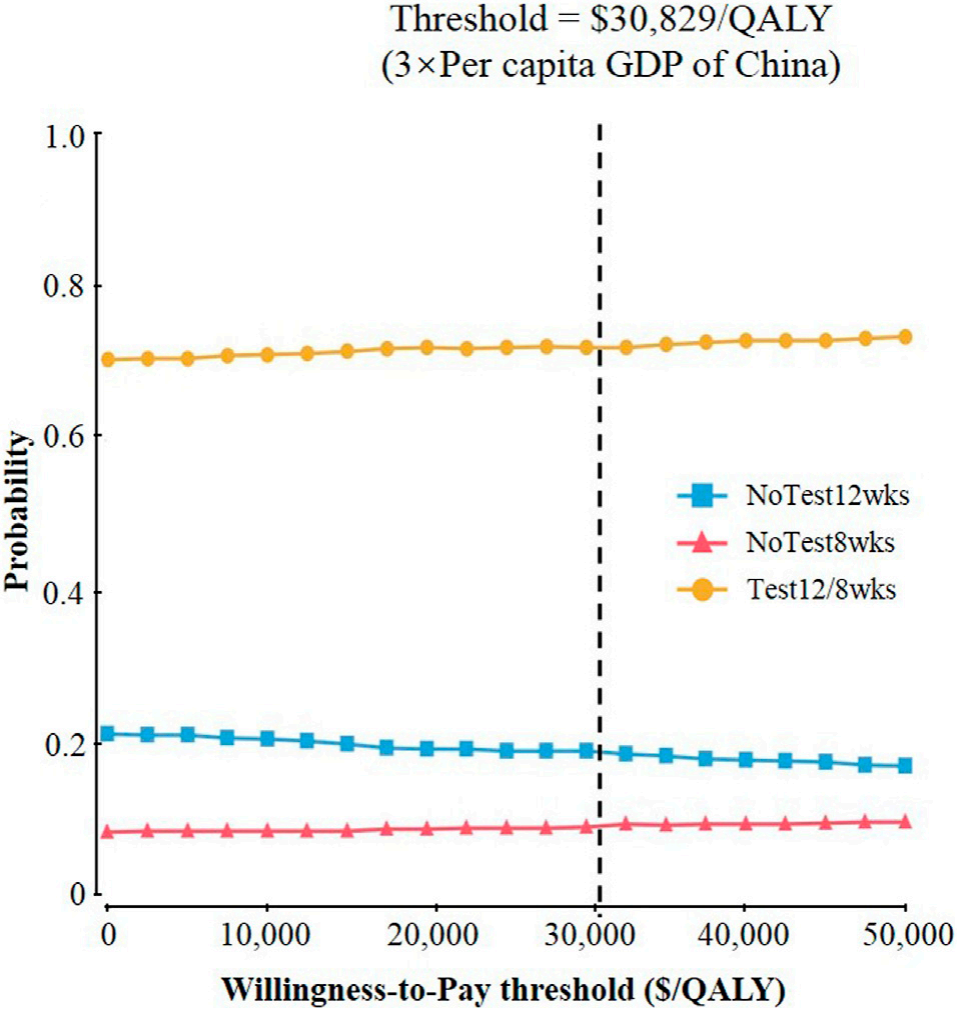
Results of probabilistic sensitivity analyses. The cost-effectiveness acceptability curves represent probabilities of being cost-effective achieved by different competing strategies at willingness-to-pay thresholds for patients with chronic hepatitis C virus genotype 1b infection.

Probabilistic sensitivity analysis confirmed the aforementioned results (Figure 4). At a threshold of USD 30,829/QALY, the probability that Test 12/8 weeks would be cost-effective was approximate 70%; While the corresponding probabilities of NoTest 12 weeks and NoTest 8 weeks were lower than 20%.

### Scenario Analyses

Table 3 showed the outcome of relevant additional scenario analyses. The first and second alternative scenario, in which target patients entered the model in the F4 state only or the F0–F3 states, provided similar results to the base case. If we assumed that the drug costs of second-line reduced by 85%, which based on the fact that the three types of hepatitis C drugs fell by more than 85% on average during last year’s national health-care price talks, the results revealed that Test 12/8 weeks was found to be dominant (lower costs and greater QALYs) compared with other two strategies. When switching the second-line drug regimen from SOF/VEL/VOX to GLE/PIB, the results demonstrated that Test 12/8 weeks was found to be dominant, while NoTest 8 weeks was dominated (less effective and more costly). If we assumed that the SVR rate of NoTest 8 weeks increased to 95%, NoTest 8 weeks strategy becomes a dominant option.

**TABLE 3:** Summary of cost and benefit in the additional scenario analysis.

	Cost (2019 USD)	Effectiveness (QALYs)	ICER (USD/QALY gained)
(A) Target patients entered the model in the F4 state only			
NoTest 12 weeks	31,790.90	11.517	Comparator
NoTest 8 weeks	32,573.14	11.512	Dominated
Test 12/8 weeks	31,801.61	11.518	10,710
(B) Target patients entered the model in the F0–F3 states			
NoTest 12 weeks	3,164.11	13.450	Comparator
NoTest 8 weeks	3,966.18	13.449	Dominated
Test 12/8 weeks	3,177.69	13.452	6,790
(C) Drug costs of second-line reduced by 85%			
NoTest 12 weeks	3,579.75	13.402	Comparator
NoTest 8 wks	3,466.39	13.400	56,680
Test 12/8 weeks	3,422.95	13.404	Cost-saving
(D) Drug regimen for second-line therapy switched to GLE/PIB			
NoTest 12 weeks	3,632.14	13.403	Comparator
NoTest 8 weeks	3,697.31	13.402	Dominated
Test 12/8 weeks	3,506.38	13.405	Cost-saving
(E) SVR rate of NoTest 8 weeks increased to 95%			
NoTest 12 weeks	3,884.73	13.402	Comparator
NoTest 8 weeks	3,790.70	13.404	Cost-saving
Test 12/8 weeks	3,898.23	13.404	6,750

†Abbreviations: USD, United states Dollars; QALYs, quality-adjusted life years; ICER, incremental cost-effectiveness ratio; F0–F4, Metavir fibrosis score; SVR, sustained virologic response; GLE/PIB, glecaprevir/pibrentasvir.

## Discussion

In this research, we used a modeling approach to examine the cost-effectiveness of oral EBR/GZR treatment based on the presence of NS5A resistance and different treatment length in patients with HCV GT 1b infection in China. Our base case analysis revealed that compared with NoTest 12 weeks, prior assessment of NS5A sensitivity followed by optimizing treatment duration was an economic strategy at a WTP threshold of USD 30,829/QALY. Furthermore, NoTest 8 weeks treatment was considered to be economically dominant with the SVR rate increased to 95%.

Personalized medicine is the new paradigm of modern medicine, aiming to predict the risk and treatment of disease on the basis of a person's genetic profile. However, the cost of the baseline screening for NS5A RAS in EBR/GZR-treated patients may lead to unfavorable economic outcomes. In addition, the availability of screening for the presence of baseline RAS varies from place to place, providers are not familiar with the test, and there is no standardized assay for the determination of resistance.

Several other modeling studies have shown that treatment with EBR/GZR is a good use of limited healthcare resources in the United States and European countries. For example, Corman and others 2017 study (Chhatwal et al., 2015; Chahal et al., 2016; Chen et al., 2016; Chen and Chen, 2017) in the US setting compared EBR/GZR regimens with ledipasvir/sofosbuvir (LDV/SOF), ombitasvir/paritaprevir/ritonavir plus dasabuvir ± ribavirin (3D ± RBV), and sofosbuvir/velpatasvir (SOF/VEL) in patients with chronic hepatitis C GT-1 infection. They showed that EBR/GZR ± RBV was the economically dominant regimen for treating GT1a noncirrhotic and GT1b TN cirrhotic patients, and was cost-saving in all other populations. Rolli and others (Rolli et al., 2018) demonstrated that EBR/GZR is a high-value strategy compared with sofosbuvir plus PR in the Italian scenario. Unlike other studies, Elbasha and others (Elbasha E. et al., 2017) and Maunoury and others (Maunoury et al., 2018) focused on cost-effectiveness of EBR/GZR in patients with two diseases (chronic hepatitis C and chronic kidney disease) and provided very similar findings that EBR/GZR can be considered cost-effective in the United States and France.

As the launch of DAAs in China, there are some studies estimating the cost-effectiveness of EBR/GZR in Chinese patients infected with HCV (Chen et al., 2018; Yun et al., 2020; Yuen et al., 2021). Chen et al. (2018) found treatment with EBR/GZR was the economically dominant regimen for Chinese patients with chronic HCV GT 1b infection in comparison with daclatasvir plus asunaprevir (DCV + ASV) regardless of cirrhosis status or treatment history. Yuen et al.’s study (Yuen et al., 2021) demonstrated that EBR/GZR was the least costly DAA and economically dominant over most other DAAs. Yun et al. (2020) found SOF/VEL was not cost-effective with the incremental cost-utility ratios of US$369,627 per QALY compared with EBR/GZR. None of these studies focused on baseline testing for RASs in China as we did. In the United States, a paper published by Elbasha and others (Elbasha EH. et al., 2017) evaluated the cost-effectiveness of baseline testing in GT 1a-infected subjects. Nevertheless, the authors treated patients for 12 weeks if no NS5A RASs were present at baseline and 16 weeks otherwise. To our knowledge, economic assessments for EBR/GZR short-duration therapy are limited. Hence, our study confirms and extends prior work by incorporating baseline testing for resistance to NS5A, and analyzing the cost-effectiveness of the shortened 8-weeks treatment without testing.

There are limitations associated with the current study. First, like all models, generalizability of the results to the target population of other races/ethnicities or in other countries may be uncertain due to the heterogeneity of payer perspectives and the country-specific epidemiologic data used. Moreover, although much of the data constructed for the model were collected from Chinese context, some data were also extrapolated from other countries. An updated pharmacoeconomic analysis should be explored when these data are available in Chinese setting. Second, some of the data used are based on small subgroups of patients extracted from a larger-scale randomized clinical trial. Small numbers, coupled with the post hoc analysis may lead to bias in the estimates. Third, our analysis did not incorporate costs associated with treatment-related adverse events, because the overall safety profile of EBR/GZR is favorable. In addition, we did not evaluate the cost-effectiveness of testing for other resistance variants exist (e.g., NS5B and NS3) and the performance characteristics (e.g., sensitivity, specificity) of a diagnostic test, but could be examined with further research. Finally, we did not perform a budget impact analysis to assess the potential cost savings of this strategy. Due to the enormous amount of chronic hepatitis C cases in China, the financial burdens for the health care system might be heavy.

Despite these limitations, our research has several key strengths. First, to the best of our knowledge, this is the first pharmacoeconomic analysis that assessed cost-effectiveness of the shortened 8-weeks treatment duration for EBR/GZR, which not only to address the burden of high treatment costs that arise with longer treatment durations but also better deliver care to more patients quicker. Second, we incorporated baseline testing for resistance to NS5A inhibitor-containing regimens in GT 1b patients in China, which are considered to be critical impede to NS5A inhibitor-resistant patients, into our hybrid modeling and extensively examined how these changes in parameters have an impact on model results.

In conclusion, Test 12/8 weeks strategy is considered to be a high-value therapy option for patients with chronic hepatitis C virus GT 1b infection from the perspective of Chinese health care payer, and NoTest 8 weeks treatment was shown to be dominant with the SVR rate increased to 95%.

## Data Availability

The original contributions presented in the study are included in the article/Supplementary Material, further inquiries can be directed to the corresponding authors.
